# Asian bat skin mycobiome maintains defense against *Pseudogymnoascus destructans* during hibernation

**DOI:** 10.1128/spectrum.00611-25

**Published:** 2025-07-23

**Authors:** Xinzhao Tong

**Affiliations:** 1 Department of Biosciences and Bioinformatics, School of Science, Xi’an Jiaotong-Liverpool University, Suzhou, China; China Agricultural University, Beijing, China

**Keywords:** white-nose syndrome, *Pseudogymnoascus destructans*, East Asian bats, skin mycobiome, hibernation

## Abstract

Since its discovery in 2006, white-nose syndrome (WNS), caused by the fungus *Pseudogymnoascus destructans*, has devastated North American bat populations (W. F. Frick, J. F. Pollock, A. C. Hicks, K. E. Langwig, et al., Science 329:679–682, 2010, https://doi.org/10.1126/science.1188594). In contrast, certain Asian bats, such as *Rhinolophus ferrumequinum* (greater horseshoe bat), exhibit significant resistance, prompting research into the mechanisms behind this resilience. A recent study by H. Leng, A. Li, Z. Li, J. R. Hoyt, et al. (Microbiol Spectr 13:e02233-24, 2025, https://doi.org/10.1128/spectrum.02233-24) explored fungal community dynamics on *R. ferrumequinum* skin and in their cave environment during hibernation using ITS amplicon sequencing and ecological modeling. The authors identified *Debaryomyces* as a dominant, potentially protective skin-associated fungus. This work provides fresh insights into microbe-mediated resistance in bats and highlights microbiome-based strategies as a promising avenue for WNS mitigation.

## COMMENTARY

White-nose syndrome (WNS), caused by the psychrophilic fungus *Pseudogymnoascus destructans*, thrives in the cold, moist conditions of hibernating bats’ winter roosts, posing a severe threat to North American populations. Since the pathogen’s arrival, these bats have suffered dramatic declines, while Asian species like *Myotis pilosus* and *Rhinolophus ferrumequinum* remain largely unaffected. This resilience suggests inherent protective adaptations, driving efforts to uncover their basis. Previous research on *M. pilosus* identified 12 bacterial species that inhibit *P. destructans* via volatile compounds (1–3). Building on this, Leng et al. (2) shifted focus to fungal communities (i.e., mycobiome), investigating how the skin mycobiome of *R. ferrumequinum* might resist WNS during hibernation.

In their study, Leng and colleagues (2) conducted a longitudinal investigation of skin- and cave-associated fungal communities in *R. ferrumequinum* inhabiting Gezi Cave, Jilin Province, China. Samples were collected across early, middle, and late hibernation (December 2017, January 2018, and April 2018), and ITS1 amplicon sequencing was used to characterize fungal assemblages. They found that *P. destructans* levels on bat skin remained low despite its greater prevalence in the cave environment. This disparity points to the skin as a potential biological barrier to fungal pathogen colonization. Neutral community models further revealed that most fungal taxa dispersed stochastically between cave and skin environments. However, *P. destructans* showed limited transfer to bat skin, and there was no cumulative increase in its abundance over time. This contrasts with a previous North American study (4), where naive little brown bats became infected after 8 weeks in contaminated hibernacula, underscoring the potential robustness of resistance in *R. ferrumequinum*.

A key finding is the dominance of *Debaryomyces*, a yeast ~20 times more abundant on bat skin than in the cave, alongside filamentous fungi such as *Penicillium*. Additionally, the relative abundance of *Debaryomyces* rose from 10% to over 25% after 1 month of hibernation and remained high until the period ended. The authors propose these fungal taxa as a potential mediator of resistance by producing antifungal metabolites, echoing patterns in WNS-resistant North American bats (5) and bacterial communities in *M. pilosus* (3). Moreover, the absence of significant alpha or beta diversity differences between infected and uninfected bats suggests that a stable, resilient skin mycobiome sustains antifungal protection throughout hibernation, limiting *P. destructans* to low levels even in infected individuals.

By integrating temporal (i.e., hibernation stages) and spatial (i.e., skin vs cave) analyses with ecological modeling, this study reveals a persistent, protective mycobiome during a vulnerable period for bats. While limited to a single hibernaculum and observational in scope, these constraints do not diminish its value; they instead set the stage for targeted follow-ups. For example, *in vitro* or *in vivo* assays could validate the antifungal role of *Debaryomyces*, while broader sampling might confirm whether this resistance is present across hibernation phases or East Asian bat populations.

The research also opens the door to global comparisons. For example, controlled exposure experiments—where Asian bats are introduced into contaminated North American hibernacula—could reveal microbiome-driven resistance mechanisms unique to Asian species. Furthermore, multi-omics approaches like metagenomics and metabolomics would allow functional characterization of anti-*P*. *destructans* metabolites, while network analyses could clarify microbial interactions across domains that bolster resistance.

Ultimately, this study suggests a model in which bat skin fungi, shaped by environmental exposure and stochastic colonization, may play a key role in host defense. If *Debaryomyces* is confirmed as a protective taxon across bat species and continents, microbial interventions, such as the strategic introduction of beneficial fungi into hibernacula, could offer innovative solutions for WNS mitigation.

In summary, Leng and colleagues (2) provide a timely and impactful contribution to WNS research by highlighting the role of the skin mycobiome—particularly *Debaryomyces*—in resisting fungal infection during bat hibernation. Their work advances our understanding of microbiome-host-pathogen interactions and introduces new possibilities for microbe-based conservation strategies. Future research incorporating functional validation, expanded geographic sampling, and multi-omics will be crucial to harness these insights for practical application. This study reinforces the notion that microbial communities are not just ecological bystanders but active allies in the fight against wildlife disease.

## References

[1] Frick WF, Pollock JF, Hicks AC, Langwig KE, Reynolds DS, Turner GG, Butchkoski CM, Kunz TH. 2010. An emerging disease causes regional population collapse of a common North American bat species. Science 329:679–682. doi:10.1126/science.1188594 20689016

[2] Leng H, Li A, Li Z, Hoyt JR, Dai W, Xiao Y, Feng J, Sun K. 2025. Variation and assembly mechanisms of Rhinolophus ferrumequinum skin and cave environmental fungal communities during hibernation periods. Microbiol Spectr 13:e02233-24. doi:10.1128/spectrum.02233-24 39846756 PMC11878040

[3] Lu Y, Ren H, Li Z, Leng H, Li A, Dai W, Huang L, Feng J, Sun K. 2024. Microbiota diversity and anti-Pseudogymnoascus destructans bacteria isolated from Myotis pilosus skin during late hibernation. Appl Environ Microbiol 90:e0069324. doi:10.1128/aem.00693-24 39058040 PMC11337810

[4] Hicks AC, Darling SR, Flewelling JE, von Linden R, Meteyer CU, Redell DN, White JP, Redell J, Smith R, Blehert DS, Rayman-Metcalf NL, Hoyt JR, Okoniewski JC, Langwig KE. 2023. Environmental transmission of Pseudogymnoascus destructans to hibernating little brown bats. Sci Rep 13:4615. doi:10.1038/s41598-023-31515-w 36944682 PMC10030556

[5] Vanderwolf KJ, Campbell LJ, Goldberg TL, Blehert DS, Lorch JM. 2021. Skin fungal assemblages of bats vary based on susceptibility to white-nose syndrome. ISME J 15:909–920. doi:10.1038/s41396-020-00821-w 33149209 PMC8027032

